# The genome sequence of common fleabane, *Pulicaria dysenterica* (L.) Bernh. (Asteraceae)

**DOI:** 10.12688/wellcomeopenres.20003.1

**Published:** 2023-10-12

**Authors:** Maarten J. M. Christenhusz, Michael F. Fay, Royal Botanic Gardens Kew Genome Acquisition Lab, Darwin Tree of Life Barcoding collective, Plant Genome Sizing collective, Wellcome Sanger Institute Tree of Life programme, Wellcome Sanger Institute Scientific Operations: DNA Pipelines collective, Tree of Life Core Informatics collective, Darwin Tree of Life Consortium

**Affiliations:** 1 Royal Botanic Gardens Kew, Richmond, England, UK

**Keywords:** Pulicaria dysenterica, common fleabane, genome sequence, chromosomal, Asterales

## Abstract

We present a genome assembly from an individual *Pulicaria dysenterica* (common fleabane; Tracheophyta; Magnoliopsida; Asterales; Asteraceae). The genome sequence is 833.2 megabases in span. Most of the assembly is scaffolded into 9 chromosomal pseudomolecules. The mitochondrial and plastid genomes were assembled and have lengths of 375.47 kilobases and 150.94 kilobases respectively.

## Species taxonomy

Eukaryota; Viridiplantae; Streptophyta; Streptophytina; Embryophyta; Tracheophyta; Euphyllophyta; Spermatophyta; Magnoliopsida; Mesangiospermae; eudicotyledons; Gunneridae; Pentapetalae; asterids; campanulids; Asterales; Asteraceae; Asteroideae; Inuleae; Inulinae; *Pulicaria*; *Pulicaria dysenterica* (L.) Bernh. (NCBI:txid56535).

## Background

*Pulicaria dysenterica* belongs to Asteraceae and is a rhizomatous, perennial herb of damp or wet, open habitats. It can form dense clusters, especially in marshy places like fen-meadows, reed beds, dune slacks, wet hollows, and the edges of lakes, rivers, canals, streams, ditches and seepages on sea cliffs. It is also found at the edges of damp woodland and roadside verges, and it is sometimes cultivated along pond edges in gardens, where it prefers sun and plenty of water. It can be found from sea level to 325 m elevation and is a Eurasian Southern-temperate element in the flora, reaching its northern limit in eastern Denmark. It is common in England, Wales and Ireland, but rare in Scotland ( OABIF, 2022).

Plants are hairy and grow to about 60 cm tall with alternate leaves that clasp the stem. The golden-yellow flowers are arranged in dense heads with a centre of up to 100 bisexual disc florets surrounded by up to 30 narrow ray florets, which are female. In fruit, the flower heads reflex exposing the fluffy pappus. Seeds are dispersed by wind.

The leaves of common fleabane are the main food for the fleabane tortoise beetle ( *Cassida murraea*), and the larvae of the dusky plume ( *Oidaematophorus lithodactyla*), a micromoth, also feed on leaves and shoots ( Kimber, 2022; Salisbury, 2004). The larvae of three other micromoths also feed on fleabane: larvae of the light fleabane neb ( *Ptocheuusa paupella*) and dark fleabane neb ( *Apodia bifractella*) feed on the seeds and flowers, and the larvae of the fleabane fanner ( *Digitivalva pulicariae*) mine the leaves ( Kimber, 2022).

Both the generic name *Pulicaria* and common name fleabane refer to its former use as an insectifuge ( *Pulicaria* is derived from *Pulex*, a genus of fleas). Dried stems were burned to rid linen of fleas and other insects. The salty-astringent juice from the fresh plant was formerly used for various maladies including dysentery, hence its specific name ( Horwood, 1919). A recent study has demonstrated biological activity against a range of bacteria, potentially supporting the use against dysentery ( Radulović *et al.*, 2022).

This is the first complete genome sequence of *Pulicaria*, and it will contribute to the importance of Asteraceae studies, such as those involving genomics and genome size ( Palazzesi *et al.*, 2022).

## Genome sequence report

**Figure 1.**
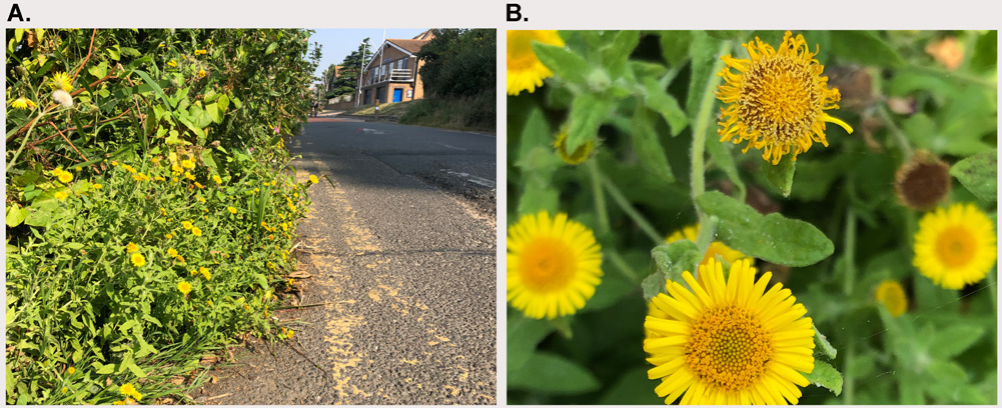
Photographs of the *Pulicaria dysenterica* (daPulDyse1) specimen used for genome sequencing. **A**. Habitat along Lower Ham Road in Kingston upon Thames. **B**. Detail of inflorescences.

The genome was sequenced from one *Pulicaria dysenterica* specimen ( Figure 1) collected from Kingston upon Thames, Surrey, UK (51.42, –0.31). Using flow cytometry, the genome size (1C-value) was estimated to be 1.10 pg, equivalent to 1,070 Mb. A total of 28-fold coverage in Pacific Biosciences single-molecule HiFi long reads and 35-fold coverage in 10X Genomics read clouds was generated. Primary assembly contigs were scaffolded with chromosome conformation Hi-C data. Manual assembly curation corrected 30 missing joins or mis-joins and removed 6 haplotypic duplications, increasing the assembly length by 8.43%, reducing the scaffold number by 11.43%, and decreasing the scaffold N50 by 45.48%.

**Figure 2.**
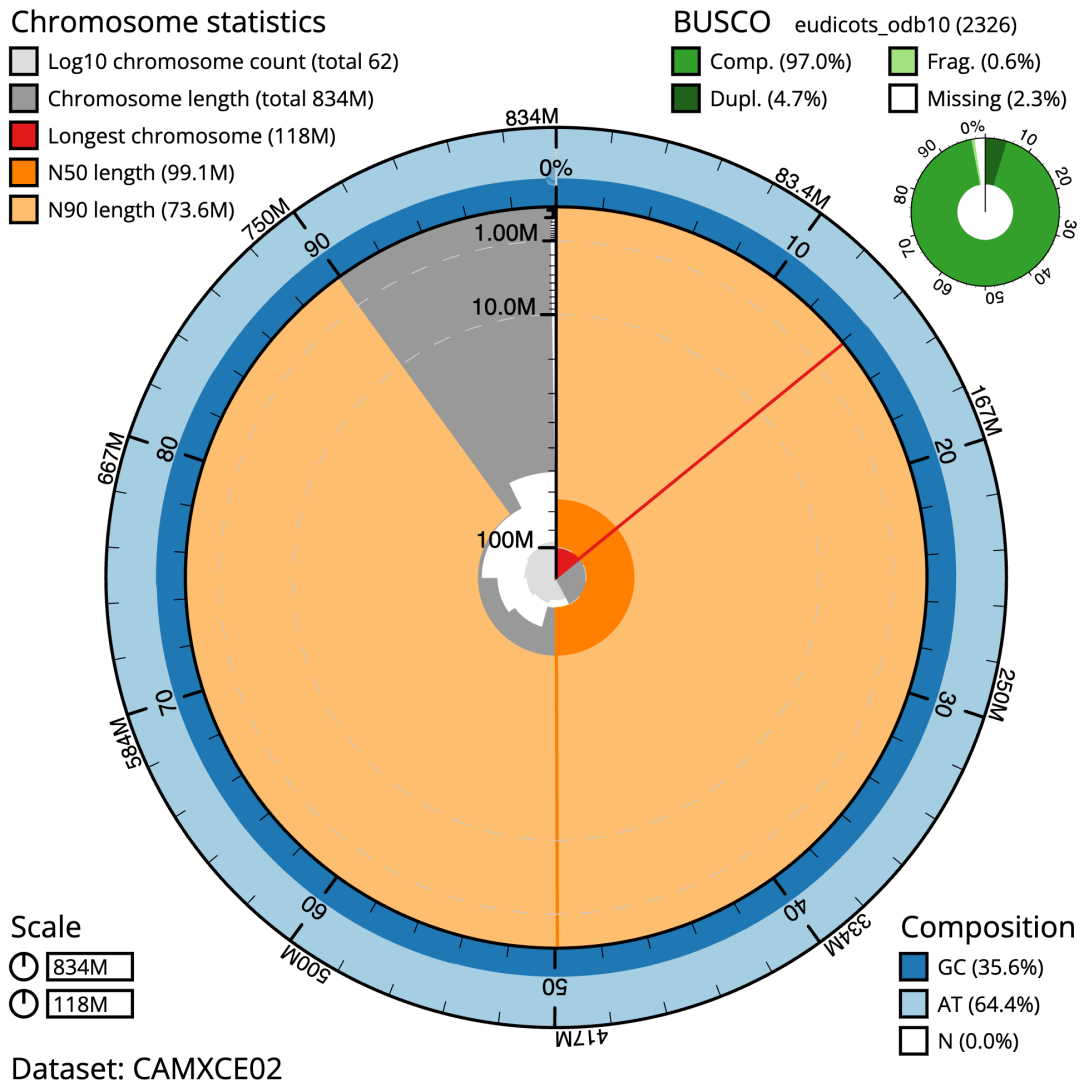
Genome assembly of *Pulicaria dysenterica*, daPulDyse1.2: metrics. The BlobToolKit Snailplot shows N50 metrics and BUSCO gene completeness. The main plot is divided into 1,000 size-ordered bins around the circumference with each bin representing 0.1% of the 833,756,611 bp assembly. The distribution of scaffold lengths is shown in dark grey with the plot radius scaled to the longest scaffold present in the assembly (118,229,540 bp, shown in red). Orange and pale-orange arcs show the N50 and N90 scaffold lengths (99,126,001 and 73,589,420 bp), respectively. The pale grey spiral shows the cumulative scaffold count on a log scale with white scale lines showing successive orders of magnitude. The blue and pale-blue area around the outside of the plot shows the distribution of GC, AT and N percentages in the same bins as the inner plot. A summary of complete, fragmented, duplicated and missing BUSCO genes in the eudicots_odb10 set is shown in the top right. An interactive version of this figure is available at https://blobtoolkit.genomehubs.org/view/Pulicaria%20dysenterica/dataset/CAMXCE02/snail.

The final assembly has a total length of 833.2 Mb in 60 sequence scaffolds with a scaffold N50 of 99.1 Mb ( Table 1). Most (99.66%) of the assembly sequence was assigned to 9 chromosomal-level scaffolds. Chromosome-scale scaffolds confirmed by the Hi-C data are named in order of size ( Figure 2– Figure 5; Table 2). While not fully phased, the assembly deposited is of one haplotype. Contigs corresponding to the second haplotype have also been deposited. The mitochondrial and plastid genomes were also assembled and can be found as contigs within the multifasta file of the genome submission.

**Table 1.** Genome data for *Pulicaria dysenterica*, daPulDyse1.2.

Project accession data		
Assembly identifier	daPulDyse1.2	
Species	*Pulicaria dysenterica*	
Specimen	daPulDyse1	
NCBI taxonomy ID	56535	
BioProject	PRJEB50479	
BioSample ID	SAMEA7522051	
Isolate information	daPulDyse1, leaves and flowers (DNA sequencing, Hi-C scaffolding and RNA sequencing)	
Assembly metrics *		*Benchmark*
Consensus quality (QV)	52.1	*≥ 50*
*k*-mer completeness	99.97	*≥ 95%*
BUSCO **	C:97.0%[S:92.3%,D:4.7%], F:0.6%,M:2.3%,n:2,326	*C ≥ 95%*
Percentage of assembly mapped to chromosomes	99.66%	*≥ 95%*
Sex chromosomes	-	*localised* *homologous pairs*
Organelles	Mitochondrial and plastid genomes assembled	*complete single* *alleles*
Raw data accessions		
PacificBiosciences SEQUEL II	ERR8482051, ERR8482053, ERR8482052	
10X Genomics Illumina	ERR8373772, ERR8373773, ERR8373771, ERR8373774	
Hi-C Illumina	ERR8373770	
PolyA RNA-Seq Illumina	ERR11641091	
Genome assembly		
Assembly accession	GCA_947179395.2	
*Accession of alternate* *haplotype*	GCA_947179335.2	
Span (Mb)	833.2	
Number of contigs	129	
Contig N50 length (Mb)	22.1	
Number of scaffolds	60	
Scaffold N50 length (Mb)	99.1	
Longest scaffold (Mb)	118.2	

* Assembly metric benchmarks are adapted from column VGP-2020 of “Table 1: Proposed standards and metrics for defining genome assembly quality” from ( Rhie *et al.*, 2021). ** BUSCO scores based on the eudicots_odb10 BUSCO set using v5.3.2. C = complete [S = single copy, D = duplicated], F = fragmented, M = missing, n = number of orthologues in comparison. A full set of BUSCO scores is available at https://blobtoolkit.genomehubs.org/view/Pulicaria%20dysenterica/dataset/CAMXCE02/busco.

**Figure 3.**
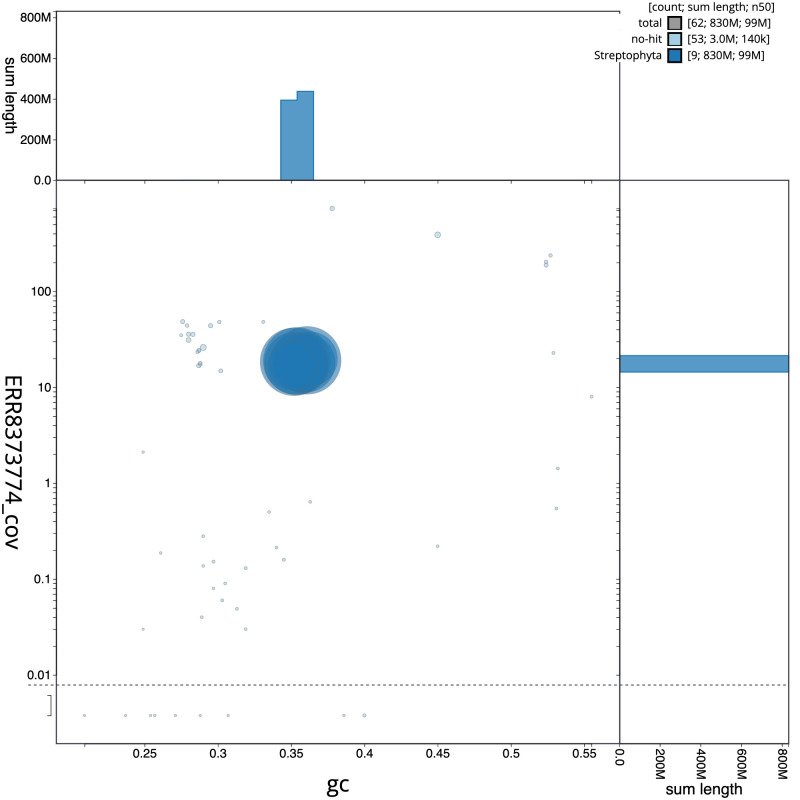
Genome assembly of *Pulicaria dysenterica*, daPulDyse1.2: BlobToolKit GC-coverage plot. Scaffolds are coloured by phylum. Circles are sized in proportion to scaffold length. Histograms show the distribution of scaffold length sum along each axis. An interactive version of this figure is available at https://blobtoolkit.genomehubs.org/view/Pulicaria%20dysenterica/dataset/CAMXCE02/blob.

**Figure 4.**
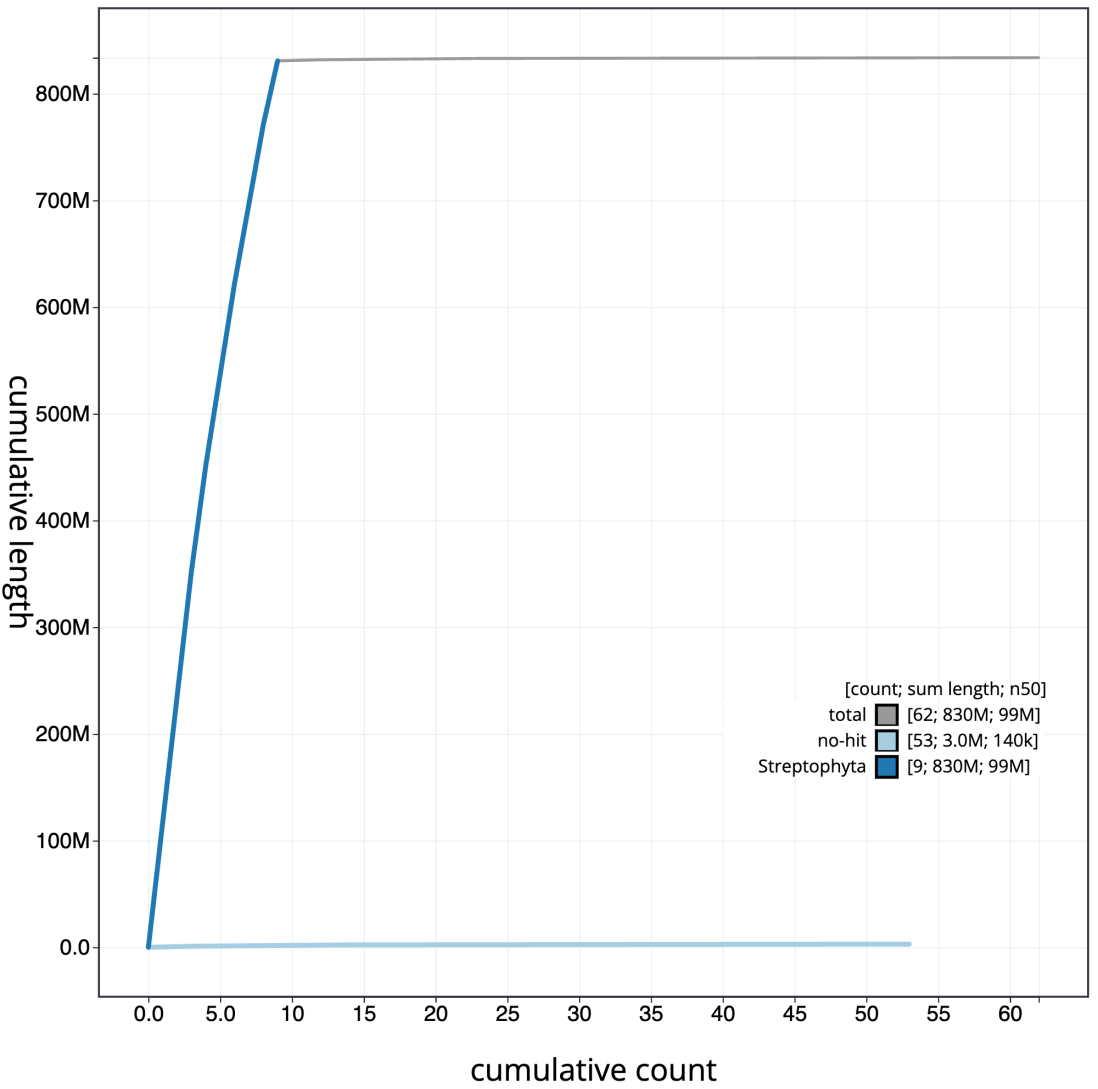
Genome assembly of *Pulicaria dysenterica*, daPulDyse1.2: BlobToolKit cumulative sequence plot. The grey line shows cumulative length for all scaffolds. Coloured lines show cumulative lengths of scaffolds assigned to each phylum using the buscogenes taxrule. An interactive version of this figure is available at https://blobtoolkit.genomehubs.org/view/Pulicaria%20dysenterica/dataset/CAMXCE02/cumulative.

**Figure 5.**
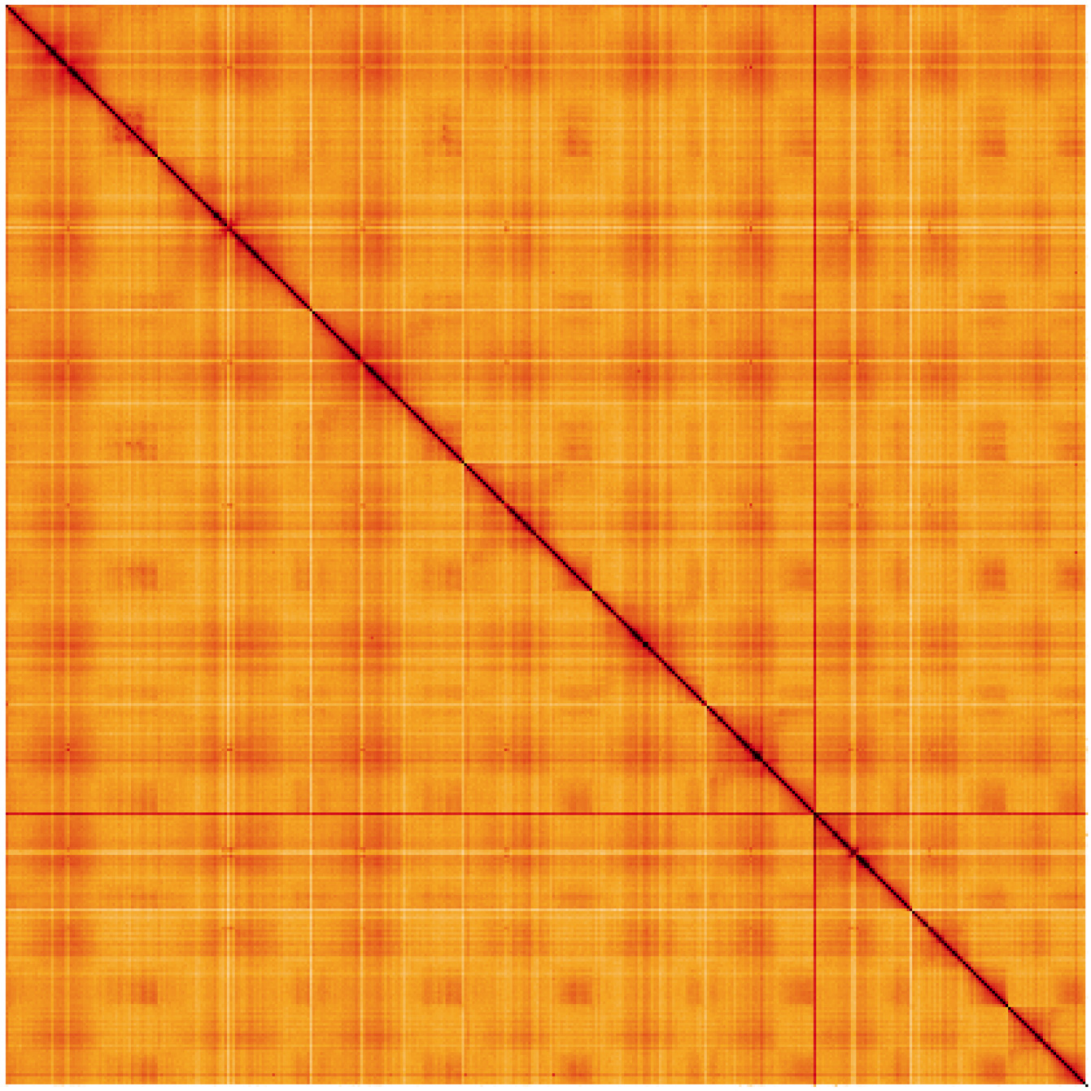
Genome assembly of *Pulicaria dysenterica*, daPulDyse1.2: Hi-C contact map of the daPulDyse1.2 assembly, visualised using HiGlass. Chromosomes are shown in order of size from left to right and top to bottom. An interactive version of this figure may be viewed at https://genome-note-higlass.tol.sanger.ac.uk/l/?d=JSiZBnchQRe3y3PaJKhs4Q.

**Table 2.** Chromosomal pseudomolecules in the genome assembly of *Pulicaria dysenterica*, daPulDyse1.

INSDC accession	Chromosome	Length (Mb)	GC%
OX359288.1	1	118.23	35.0
OX359289.1	2	118.0	36.0
OX359290.1	3	116.35	35.5
OX359291.1	4	99.13	35.5
OX359292.1	5	87.21	36.0
OX359293.1	6	83.17	35.5
OX359294.1	7	75.03	36.5
OX359295.1	8	73.59	35.5
OX359296.1	9	60.06	35.0
OX381670.1	MT	0.38	45.0
OX381671.1	Pltd	0.15	37.5

The estimated Quality Value (QV) of the final assembly is 52.1 with *k*-mer completeness of 99.97%, and the assembly has a BUSCO v5.3.2 completeness of 97.0% (single = 92.3%, duplicated = 4.7%), using the eudicots_odb10 reference set ( *n* = 2,326).

Metadata for specimens, spectral estimates, sequencing runs, contaminants and pre-curation assembly statistics can be found at https://links.tol.sanger.ac.uk/species/56535.

## Methods

### Sample acquisition, genome size estimation and nucleic acid extraction

Samples of an individual *Pulicaria dysenterica* (specimen ID KDTOL10042, ToLIDdaPulDyse1) was picked by hand along the River Thames in Canbury Gardens, Kingston upon Thames, Surrey (latitude 51.42, longitude –0.31) on 2020-08-12. The specimen was collected and identified by Maarten J. M. Christenhusz (Royal Botanic Gardens, Kew) and frozen at –80°C.

The genome size was estimated by flow cytometry using the fluorochrome propidium iodide and following the ‘one-step’ method as outlined in Pellicer *et al*. (2021). Specifically for this species, General Purpose Buffer (GPB) supplemented with 3% PVP and 0.08% (v/v) beta-mercaptoethanol was used for isolation of nuclei ( Loureiro *et al.*, 2007), and the internal calibration standard used was *Petroselinum crispum* ‘Champion Moss Curled’ with an assumed 1C-value of 2,200 Mb ( Obermayer *et al.*, 2002).

DNA was extracted at the Tree of Life laboratory, Wellcome Sanger Institute (WSI). The daPulDyse1 sample was weighed and dissected on dry ice with tissue set aside for Hi-C sequencing. Flower and leaf samples were cryogenically disrupted to a fine powder using a Covaris cryoPREP Automated Dry Pulveriser, receiving multiple impacts. High molecular weight (HMW) DNA was extracted using the Qiagen Plant MagAttract DNA extraction kit. Low molecular weight DNA was removed from a 20 ng aliquot of extracted DNA using the 0.8X AMpure XP purification kit prior to 10X Chromium sequencing; a minimum of 50 ng DNA was submitted for 10X sequencing. HMW DNA was sheared into an average fragment size of 12–20 kb in a Megaruptor 3 system with speed setting 30. Sheared DNA was purified by solid-phase reversible immobilisation using AMPure PB beads with a 1.8X ratio of beads to sample to remove the shorter fragments and concentrate the DNA sample. The concentration of the sheared and purified DNA was assessed using a Nanodrop spectrophotometer and Qubit Fluorometer and Qubit dsDNA High Sensitivity Assay kit. Fragment size distribution was evaluated by running the sample on the FemtoPulse system.

RNA was extracted from leaf tissue of daPulDyse1 in the Tree of Life Laboratory at the WSI using TRIzol, according to the manufacturer’s instructions. RNA was then eluted in 50 μl RNAse-free water and its concentration assessed using a Nanodrop spectrophotometer and Qubit Fluorometer using the Qubit RNA Broad-Range (BR) Assay kit. Analysis of the integrity of the RNA was done using Agilent RNA 6000 Pico Kit and Eukaryotic Total RNA assay.

### Sequencing

Pacific Biosciences HiFi circular consensus and 10X Genomics read cloud DNA sequencing libraries were constructed according to the manufacturers’ instructions. Poly(A) RNA-Seq libraries were constructed using the NEB Ultra II RNA Library Prep kit. DNA and RNA sequencing was performed by the Scientific Operations core at the WSI on Pacific Biosciences SEQUEL II (HiFi) and Illumina NovaSeq 6000 (RNA-Seq and 10X) instruments. Hi-C data were also generated from leaf tissue of daPulDyse1 using the Arima2 kit and sequenced on the NovaSeq 6000 instrument.

### Genome assembly, curation, and evaluation

Assembly was carried out with Hifiasm ( Cheng *et al.*, 2021) and haplotypic duplication was identified and removed with purge_dups ( Guan *et al.*, 2020). One round of polishing was performed by aligning 10X Genomics read data to the assembly with Long Ranger ALIGN, calling variants with FreeBayes ( Garrison & Marth, 2012). The assembly was then scaffolded with Hi-C data ( Rao *et al.*, 2014) using YaHS ( Zhou *et al.*, 2023). The assembly was checked for contamination and corrected as described previously ( Howe *et al.*, 2021). Manual curation was performed using HiGlass ( Kerpedjiev *et al.*, 2018) and Pretext ( Harry, 2022). The mitochondrial and chloroplast genomes were assembled using MBG from PacBio HiFi reads mapping to related genomes ( Rautiainen & Marschall, 2021). A representative circular sequence was selected for each from the graph based on read coverage.

A Hi-C map for the final assembly was produced using bwa-mem2 ( Vasimuddin *et al.*, 2019) in the Cooler file format ( Abdennur & Mirny, 2020). To assess the assembly metrics, the *k*-mer completeness and QV consensus quality values were calculated in Merqury ( Rhie *et al.*, 2020). This work was done using Nextflow ( Di Tommaso *et al.*, 2017) DSL2 pipelines “sanger-tol/readmapping” ( Surana *et al.*, 2023a) and “sanger-tol/genomenote” ( Surana *et al.*, 2023b). The genome was analysed within the BlobToolKit environment ( Challis *et al.*, 2020) and BUSCO scores ( Manni *et al.*, 2021; Simão *et al.*, 2015) were calculated.

Table 3 contains a list of relevant software tool versions and sources.

**Table 3.** Software tools: versions and sources.

Software tool	Version	Source
BlobToolKit	4.0.7	https://github.com/blobtoolkit/blobtoolkit
BUSCO	5.3.2	https://gitlab.com/ezlab/busco
FreeBayes	1.3.1-17-gaa2ace8	https://github.com/freebayes/freebayes
Hifiasm	0.15.3	https://github.com/chhylp123/hifiasm
HiGlass	1.11.6	https://github.com/higlass/higlass
Long Ranger ALIGN	2.2.2	https://support.10xgenomics.com/genome-exome/software/pipelines/latest/advanced/other-pipelines
MBG		https://github.com/maickrau/MBG
Merqury	MerquryFK	https://github.com/thegenemyers/MERQURY.FK
PretextView	0.2	https://github.com/wtsi-hpag/PretextView
purge_dups	1.2.3	https://github.com/dfguan/purge_dups
sanger-tol/genomenote	v1.0	https://github.com/sanger-tol/genomenote
sanger-tol/readmapping	1.1.0	https://github.com/sanger-tol/readmapping/tree/1.1.0
YaHS	1.0	https://github.com/c-zhou/yahs

### Wellcome Sanger Institute – Legal and Governance

The materials that have contributed to this genome note have been supplied by a Darwin Tree of Life Partner. The submission of materials by a Darwin Tree of Life Partner is subject to the **‘Darwin Tree of Life Project Sampling Code of Practice’**, which can be found in full on the Darwin Tree of Life website here. By agreeing with and signing up to the Sampling Code of Practice, the Darwin Tree of Life Partner agrees they will meet the legal and ethical requirements and standards set out within this document in respect of all samples acquired for, and supplied to, the Darwin Tree of Life Project. 

Further, the Wellcome Sanger Institute employs a process whereby due diligence is carried out proportionate to the nature of the materials themselves, and the circumstances under which they have been/are to be collected and provided for use. The purpose of this is to address and mitigate any potential legal and/or ethical implications of receipt and use of the materials as part of the research project, and to ensure that in doing so we align with best practice wherever possible. The overarching areas of consideration are:

• Ethical review of provenance and sourcing of the material

• Legality of collection, transfer and use (national and international) 

Each transfer of samples is further undertaken according to a Research Collaboration Agreement or Material Transfer Agreement entered into by the Darwin Tree of Life Partner, Genome Research Limited (operating as the Wellcome Sanger Institute), and in some circumstances other Darwin Tree of Life collaborators.

## Data Availability

European Nucleotide Archive: *Pulicaria dysenterica*. Accession number PRJEB50479; https://identifiers.org/ena.embl/PRJEB50479. ( Wellcome Sanger Institute, 2022) The genome sequence is released openly for reuse. The *Pulicaria dysenterica* genome sequencing initiative is part of the Darwin Tree of Life (DToL) project. All raw sequence data and the assembly have been deposited in INSDC databases. The genome will be annotated using available RNA-Seq data and presented through the Ensembl pipeline at the European Bioinformatics Institute. Raw data and assembly accession identifiers are reported in Table 1.

## Author information

Members of the Royal Botanic Gardens Kew Genome Acquisition Lab are listed here: https://doi.org/10.5281/zenodo.4786680.

Members of the Darwin Tree of Life Barcoding collective are listed here: https://doi.org/10.5281/zenodo.4893703.

Members of the Plant Genome Sizing collective are listed here: https://doi.org/10.5281/zenodo.7994306.

Members of the Wellcome Sanger Institute Tree of Life programme are listed here: https://doi.org/10.5281/zenodo.4783585.

Members of Wellcome Sanger Institute Scientific Operations: DNA Pipelines collective are listed here: https://doi.org/10.5281/zenodo.4790455.

Members of the Tree of Life Core Informatics collective are listed here: https://doi.org/10.5281/zenodo.5013541.

Members of the Darwin Tree of Life Consortium are listed here: https://doi.org/10.5281/zenodo.4783558.
