# Supplementary material for: The genome sequence of common fleabane, Pulicaria dysenterica (L.) Bernh. (Asteraceae)
Source: Wellcome Open Res. 2023 Oct 12;8:447. [Version 1] doi: 10.12688/wellcomeopenres.20003.1 (PMC10674090; doi:10.12688/wellcomeopenres.20003.1)
Supplement: Peer review history

# Reviewer report: Reviewer response for version 1

**DOI:** 10.21956/wellcomeopenres.22149.r69981

**Published:** 2023-11-24

**Authors:** Todd Gaines

**Affiliations:** 1 Colorado State University, Fort Collins, Colorado, USA

The article reports assembly of the genome of common fleabane, *Pulicaria dysenterica*, as part of the Darwin Tree of Life Consortium. The genome sequencing was conducted using reasonably high coverage (28X) PacBio HiFi long read sequencing combined with 10X Genomics sequencing and Hi-C chromosome conformation data. The initially assembled contigs were scaffolded into chromosome-level scaffolds (9, one for each chromosome of this species). The final assembly size was 833.2 Mb, close to the genome size estimated by flow cytometry of 1,070 Mb.

 The presentation of the genome assembly data is sufficient to assess the quality of the genome. The article provides genome assembly benchmarks used in other eukaryotic species genome assembly evaluations, and the reported genome assembly meets or exceeds all benchmarks for quality. The main assembly was not phased but haplotypic duplications were identified and removed to an alternate assembly that is also reported.

 The description of the methods is sufficient to enable assessment of the work and for someone to reproduce the work. All data have been properly provided to permanent online repositories, including the original sequence data, the main assembly, and the partial alternate haplotype. The article also reports the sampling records and processes used by DTOL to ensure compliance with ethical and legal standards.

 This article meets the expected standards for a report of a de novo plant genome assembly. My only suggestion for a change would be to provide citation(s) for the biological and morphological information provided in the first two paragraphs of the background section.

Are sufficient details of methods and materials provided to allow replication by others?

Yes

Is the rationale for creating the dataset(s) clearly described?

Yes

Are the datasets clearly presented in a useable and accessible format?

Yes

Are the protocols appropriate and is the work technically sound?

Yes

Reviewer Expertise:

Plant molecular biology, plant genomics

I confirm that I have read this submission and believe that I have an appropriate level of expertise to confirm that it is of an acceptable scientific standard.

# Reviewer report: Reviewer response for version 1

**DOI:** 10.21956/wellcomeopenres.22149.r69129

**Published:** 2023-11-06

**Authors:** Ayoob Obaid Alfalahi

**Affiliations:** 1 University of Anbar, Anbar, Iraq

Thank you for suggesting me to review the attached manuscript.

 I found the article is very interesting through which authors tried to employ modern molecular techniques to determine the whole genome sequence of *Pulicaria dysenterica* (common fleabane; Tracheophyta; Magnoliopsida; Asterales; Asteraceae). The mitochondrial and plastid genomes of fleabane were assembled too.

 However, after thoroughly reading the manuscript, I'm sending the following comments:

1. The introduction doesn’t provide sufficient background and doesn’t include all up-to-date relevant references. Also, the main objectives should be included (e.g. why it is important to complete the genome sequence for this plant species).
2. The methods can be further detailed; however, before it was diluted to the final work concentration, the quality and quantity of the extracted DNA and RNA should be indicated! Also, author may clarify whether the collected plant sample was free pest infections or it was not. What was the weight of plant material used to extract the DNA?
3. Close images to the entire plant sample will be more valuable in describing the plant morphology; especially in such important genomic studies.
4. I think it is important to indicate the origin and the manufactured company of the used materials and instruments.

I wish authors will be able to use these comments to improve their article.

Are sufficient details of methods and materials provided to allow replication by others?

Partly

Is the rationale for creating the dataset(s) clearly described?

Yes

Are the datasets clearly presented in a useable and accessible format?

Yes

Are the protocols appropriate and is the work technically sound?

Yes

Reviewer Expertise:

Plant Biotechnology

I confirm that I have read this submission and believe that I have an appropriate level of expertise to confirm that it is of an acceptable scientific standard.
